# Novel therapeutic choices in immune aplastic anemia

**DOI:** 10.12688/f1000research.22214.1

**Published:** 2020-09-10

**Authors:** Phillip Scheinberg

**Affiliations:** 1 Division of Hematology, Hospital A Beneficência Portuguesa, São Paulo, Brazil

**Keywords:** aplastic anemia, bone marrow failure, pancytopenia, antithymocyte globulin

## Abstract

Aplastic anemia (AA) in its severe form has historically been associated with high mortality. With limited supportive care and no effective strategy to reverse marrow failure, most patients diagnosed with severe AA (SAA) died of pancytopenia complications. Since the 1970s, hematopoietic stem cell transplantation (HSCT) and immunosuppressive therapy (IST) have changed SAA's natural history by improving marrow function and pancytopenia. Standard IST with horse anti-thymocyte globulin plus cyclosporine produces a hematologic response rate of 60 to 70%. In the long term, about one-third of patients relapse, and 10 to 15% can develop cytogenetic abnormalities. Outcomes with either HSCT or IST are similar, and choosing between these modalities relies on age, availability of a histocompatible donor, comorbidities, and patient preference. The introduction of eltrombopag, a thrombopoietin receptor agonist, improved SAA outcomes as both salvage (second-line) and upfront therapy combined with IST. As a single agent, eltrombopag in doses up to 150 mg daily improved cytopenias in 40 to 50% in those who failed initial IST, which associated with higher marrow cellularity, suggesting a pan-stimulatory marrow effect. When eltrombopag was combined with IST as upfront therapy, overall (about 90%) and complete responses (about 50%) were higher than observed extensively with IST alone of 65% and 10%, respectively. Not surprisingly, given the strong correlation between hematologic response rates and survival in SAA, most (>90%) were alive after a median follow-up of 18 months. Longer follow-up and real-word data continue to confirm the activity of this agent in AA. The use of eltrombopag in different combinations and doses are currently being explored. The activity of another thrombopoietin receptor agonist in AA, romiplostim, suggests a class effect. In the coming years, the mechanisms of their activity and the most optimal regimen are likely to be elucidated.

The initial treatment choice is an important decision point in the management of aplastic anemia (AA), especially in its severe form. The progression of pancytopenia can be gradual but often presents acutely in a patient who weeks earlier was otherwise doing well. In these cases, the clinical presentation is associated with complications of low blood counts, namely infections (from neutropenia), weakness (from anemia), or bleeding (from thrombocytopenia) or a combination of these. These patients often are admitted for transfusion support and evaluation. A hypocellular bone marrow points to marrow failure, and if no other causes are identified in the setting of severe pancytopenia, severe AA (SAA) is diagnosed. Exposures to drugs, chemicals, and toxins often are interrogated but rarely are the direct cause of marrow failure. A history of autoimmune disease or infections (viral) may suggest an association. But the most important differential diagnosis is with myelodysplastic syndrome, which can mimic AA ^1, 2^.

Once AA is diagnosed, it is important to differentiate between congenital and immunologic forms of the disease. Younger age of onset (childhood), family history, and physical stigmata of distinct forms of hereditary AA (dyskeratosis congenita, Fanconi anemia, and Shwachman–Diamond syndrome) can be indicative of a hereditary problem ^3^. Genetics, telomere length, and clastogenic testing often are applied in this evaluation. However, older age of onset, absence of family history, and physical stigmata do not rule out alterations in the telomere length or in the genetics, which are associated with hereditary syndromes ^4, 5^. Nonetheless, most cases of AA, especially when severe, are immune-mediated and treated with either hematopoietic stem cell transplantation (HSCT) or immunosuppressive therapy (IST) ^6^. The degree of pancytopenia defines the severity of AA ^7^.

For younger (<40 years) patients with an HLA-matched sibling, allogeneic HSCT is preferred ^5, 6^. HLA disparities and the fact that risks with HSCT increase with age are reasons why IST is preferred for patients who are older than 40 or who lack a histocompatible sibling (or both) ^8^. In some settings where a matched unrelated donor (MUD) can be readily available (<6 weeks), a MUD HSCT can be considered as the first therapy in children ^9^. However, the uncertainty of finding a histocompatible donor in registries (especially in ethnic minorities), the time for identifying such a donor (in many places this can be several months), and applying supportive care only for a patient with severe pancytopenia limit the generalizability of this approach. A haploidentical platform with post-transplant cyclophosphamide has shown encouraging results in younger patients, but experience so far has relied primarily on smaller retrospective studies with limited follow-up ^10^. The optimal conditioning has not yet been defined, and to date, this approach is considered experimental and should be applied in a clinical protocol. In the aggregate, most cases of SAA worldwide are treated with non-transplant options because of the lack of a suitable HLA-matched donor, older age, comorbidities, patient choice, or lack of access to transplant. The standard IST consists of horse anti-thymocyte globulin (h-ATG) plus cyclosporine (CsA), which is associated with hematologic recovery and transfusion independence in 60 to 70% of cases ^11^. Rabbit anti-thymocyte globulin (r-ATG) is more widely available, especially outside the US, but produces inferior outcomes; hematologic recoveries occur in about 30 to 40% of cases ^11, 12^. Several attempts have been made to improve outcomes beyond h-ATG/CsA but were unsuccessful. The addition of a third drug to h-ATG/CsA, such as granulocyte colony-stimulating factor (G-CSF), mycophenolate mofetil, or sirolimus, did not improve outcomes, and substitution of h-ATG by r-ATG, cyclophosphamide, or alemtuzumab was equally ineffective because of inferior response rates or excessive toxicities or both ^1^. The strategy that has been more fruitful was not by intensifying immunosuppression but by adding a pan-stimulatory marrow agent of the class of the thrombopoietin receptor agonists (Tpo-RAs) ^13^. This class is represented by eltrombopag, romiplostim, and avatrombopag, which were developed for immune thrombocytopenia. Most of the experience with Tpo-RA in marrow failure has been with eltrombopag, which also has approval in SAA.

About 10 years ago, the first trial of eltrombopag in SAA was initiated in patients who had failed first-line therapy with IST. In a 43-patient study, hematologic response was observed in about 40 to 50% of patients with bi- and tri-lineage count recoveries observed ^14^. Eltrombopag, in these earlier studies, was given stepwise with the initial 50 mg dose that was escalated every 2 weeks up to 150 mg for a total of 16 weeks. Response usually was noted at the higher dose range. This initial cohort was expanded to 83 patients, and the latter 40 patients received eltrombopag at a starting dose of 150 mg from day 1 up to 6 months. In this 40-patient cohort, the response rate remained at about 50% but more multilineage (robust) recoveries were observed ^15^. The possibility of discontinuation of eltrombopag was noted in the earlier trials, and the robustness of blood count recovery appeared to be associated with successful discontinuation without the need for further therapy. Relapses, when they occurred, were responsive to the resumption of eltrombopag. The mechanism by which eltrombopag was active in SAA was hypothesized to be via c-mpl (the Tpo-RA receptor) signaling and stimulation of early progenitor cells which expressed the receptor. *In vitro* and *in vivo* data supported this possibility. Of concern, however, has been the possible stimulation of abnormal clones in the marrow given the temporal association between the initiation of eltrombopag and the appearance of cytogenetic abnormalities (usually in the first 6 months) ^15^. The cumulative incidence to date has not shown an increased rate of higher-risk clonal evolution, but longer follow-up is necessary to better define this risk. More recent data support that off-target effects might positively contribute to its immunomodulatory and marrow stimulatory effects ^16^. Likely, the overall net effect of target and off-target mechanisms contributes to its effectiveness.

With the single-agent activity observed in second line, eltrombopag was added to standard h-ATG/CsA in front line in subsequent studies. In a three-cohort 92-patient study, eltrombopag was added to standard h-ATG/CsA in slightly different regimens to decrease toxicity and maximize effectiveness ^17^. The combination was well tolerated and the better overall results were observed in the third cohort which had eltrombopag initiated concomitantly with h-ATG/CsA on day 1 and continued for 6 months. In this group, the overall response rate was 94% and the complete response rate was 58%, which differ from the vast historical experience of h-ATG/CsA alone of an overall response rate of 60 to 70% and a complete response rate of about 10%. Overall survival after a median follow-up of 18 months was higher than 90 to 95% given the very intimate association between hematologic response and survival after IST in SAA ^7^. Relapse in the upfront trial occurred at an unexpectedly higher rate (close to 50%), which nearly halfway into the study led to an amendment that allowed for continued long-term use of CsA beyond 6 months (instead of its discontinuation at 6 months). This led to a significant reduction of relapses to about 10 to 15%. The risk for clonal cytogenetic evolution was an important consideration in the design of the trial, prompting periodic bone marrow evaluations. Follow-up to fully determine this risk has been relatively short, although with a median follow-up of over 2 years to date, this risk has been in accordance with what has been observed historically with non-eltrombopag-containing regimens in SAA ^5^. These data led to the approval of eltrombopag in front line with IST with benefit primarily in the adult population ^5^.

The strategy of stem cell stimulation with eltrombopag in SAA had more success than other approaches of intensifying immunosuppression or stimulation of more committed marrow progenitor cells (G-CSF, erythropoietin, and so on). Alternative mechanisms of action for eltrombopag—which includes immunomodulatory effects, evasion of inhibitory signals, and iron chelation which could be beneficial in SAA—are emerging ^16, 18, 19^. Studies outside the US have confirmed the activity of eltrombopag in SAA ^20– 24^. More recently, alternative Tpo-RAs such as romiplostim have been investigated in AA as second-line therapy also showing activity ^25, 26^. It is anticipated that romiplostim will be further developed in SAA. Markers of sustained response to eltrombopag could be useful in stratifying patients who are more likely to benefit from it ^27, 28^. Longer-term follow-up and different combinations and doses are needed to better define the role and optimal delivery of the Tpo-RAs in AA in the future.

## References

[1] Scheinberg P Young NS: How I treat acquired aplastic anemia. *Blood.* 2012;120(6):1185–96. 10.1182/blood-2011-12-274019 22517900 PMC3418715

[2] Durrani J Maciejewski JP: Idiopathic aplastic anemia vs hypocellular myelodysplastic syndrome. *Hematology Am Soc Hematol Educ Program.* 2019;2019(1):97–104. 10.1182/hematology.2019000019 31808900 PMC6913491

[3] Scheinberg P: Recent Advances and Long-Term Results of Medical Treatment of Acquired Aplastic Anemia: Are Patients Cured? * Hematol Oncol Clin North Am.* 2018;32(4):609–18. 10.1016/j.hoc.2018.03.003 30047414

[4] Calado RT Young NS: Telomere diseases. *N Engl J Med.* 2009;361(24):2353–65. 10.1056/NEJMra0903373 20007561 PMC3401586

[5] Young NS: Aplastic Anemia. *N Engl J Med.* 2018;379(17):1643–56. 10.1056/NEJMra1413485 30354958 PMC6467577

[6] Bacigalupo A: How I treat acquired aplastic anemia. *Blood.* 2017;129(11):1428–36. 10.1182/blood-2016-08-693481 28096088

[7] Rosenfeld S Follmann D Nunez O: Antithymocyte globulin and cyclosporine for severe aplastic anemia: Association between hematologic response and long-term outcome. *JAMA.* 2003;289(9):1130–5. 10.1001/jama.289.9.1130 12622583

[8] Giammarco S de Latour RP Sica S: Transplant outcome for patients with acquired aplastic anemia over the age of 40: Has the outcome improved? *Blood.* 2018;131(17):1989–92. 10.1182/blood-2017-09-807859 29549172

[9] Dufour C Veys P Carraro E: Similar outcome of upfront-unrelated and matched sibling stem cell transplantation in idiopathic paediatric aplastic anaemia. A study on behalf of the UK Paediatric BMT Working Party, Paediatric Diseases Working Party and Severe Aplastic Anaemia Working Party of EBMT. *Br J Haematol.* 2015;171(4):585–94. 10.1111/bjh.13614 26223288

[10] ElGohary G El Fakih R de Latour R: Haploidentical hematopoietic stem cell transplantation in aplastic anemia: A systematic review and meta-analysis of clinical outcome on behalf of the severe aplastic anemia working party of the European group for blood and marrow transplantation (SAAWP of EBMT). *Bone Marrow Transplant.* 2020. 10.1038/s41409-020-0897-2 32346079

[11] Scheinberg P Nunez O Weinstein B: Horse versus rabbit antithymocyte globulin in acquired aplastic anemia. *N Engl J Med.* 2011;365(5):430–8. 10.1056/NEJMoa1103975 21812672 PMC3721503

[12] Marsh JC Bacigalupo A Schrezenmeier H: Prospective study of rabbit antithymocyte globulin and cyclosporine for aplastic anemia from the EBMT Severe Aplastic Anaemia Working Party. *Blood.* 2012;119(23):5391–6. 10.1182/blood-2012-02-407684 22544699

[13] Olnes MJ Scheinberg P Calvo KR: Eltrombopag and improved hematopoiesis in refractory aplastic anemia. *N Engl J Med.* 2012;367(1):11–9. 10.1056/NEJMoa1200931 22762314 PMC3422737

[14] Desmond R Townsley DM Dumitriu B: Eltrombopag restores trilineage hematopoiesis in refractory severe aplastic anemia that can be sustained on discontinuation of drug. *Blood.* 2014;123(12):1818–25. 10.1182/blood-2013-10-534743 24345753 PMC3962161

[15] Winkler T Fan X Cooper J: Treatment optimization and genomic outcomes in refractory severe aplastic anemia treated with eltrombopag. *Blood.* 2019;133(24):2575–85. 10.1182/blood.2019000478 30992268 PMC6566590

[16] Scheinberg P: Activity of eltrombopag in severe aplastic anemia. *Blood Adv.* 2018;2(21):3054–62. 10.1182/bloodadvances.2018020248 30425070 PMC6234374

[17] Townsley DM Scheinberg P Winkler T: Eltrombopag Added to Standard Immunosuppression for Aplastic Anemia. *N Engl J Med.* 2017;376(16):1540–50. 10.1056/NEJMoa1613878 28423296 PMC5548296

[18] Zhao Z Sun Q Sokoll LJ: Eltrombopag mobilizes iron in patients with aplastic anemia. *Blood.* 2018;131(21):2399–402. 10.1182/blood-2018-01-826784 29632023 PMC5969382

[19] Fattizzo B Cavallaro F Milesi G: Iron mobilization in a real life cohort of aplastic anemia patients treated with eltrombopag. *Am J Hematol.* 2019;94(9):E237–E239. 10.1002/ajh.25550 31172568

[20] Ecsedi M Lengline É Knol-Bout C: Use of eltrombopag in aplastic anemia in Europe. *Ann Hematol.* 2019;98(6):1341–50. 10.1007/s00277-019-03652-8 30915499

[21] Lengline E Drenou B Peterlin P: Nationwide survey on the use of eltrombopag in patients with severe aplastic anemia: A report on behalf of the French Reference Center for Aplastic Anemia. *Haematologica.* 2018;103(2):212–20. 10.3324/haematol.2017.176339 29170252 PMC5792265

[22] Yamazaki H Ohta K Iida H: Hematologic recovery induced by eltrombopag in Japanese patients with aplastic anemia refractory or intolerant to immunosuppressive therapy. *Int J Hematol.* 2019;110(2):187–96. 10.1007/s12185-019-02683-1 31183813

[23] Hwang YY Gill H Chan TSY: Eltrombopag in the management of aplastic anaemia: Real-world experience in a non-trial setting. *Hematology.* 2018;23(7):399–404. 10.1080/10245332.2017.1422306 29303047

[24] Konishi A Nakaya A Fujita S: Evaluation of eltrombopag in patients with aplastic anemia in real-world experience. *Leuk Res Rep.* 2019;11:11–3. 10.1016/j.lrr.2019.03.002 30911464 PMC6416522

[25] Scheinberg P: Stem cell stimulation continues to pay off in aplastic anaemia. *Lancet Haematol.* 2019;6(11):e543–e544. 10.1016/S2352-3026(19)30181-4 31474547

[26] Lee JW Lee SE Jung CW: Romiplostim in patients with refractory aplastic anaemia previously treated with immunosuppressive therapy: A dose-finding and long-term treatment phase 2 trial. *Lancet Haematol.* 2019;6(11):e562–e572. 10.1016/S2352-3026(19)30153-X 31474546

[27] Zhao X Feng X Wu Z: Persistent elevation of plasma thrombopoietin levels after treatment in severe aplastic anemia. *Exp Hematol.* 2018;58:39–43. 10.1016/j.exphem.2017.09.006 28941711 PMC5815891

[28] Fattizzo B Kulasekararaj AG Hill A: Clinical and morphological predictors of outcome in older aplastic anemia patients treated with eltrombopag. *Haematologica.* 2019;104(11):e494–e496. 10.3324/haematol.2019.216374 30890599 PMC6821617

